# Nanoscale Pathway of Modern Dolomite Formation in a Shallow, Alkaline Lake

**DOI:** 10.1021/acs.cgd.2c01393

**Published:** 2023-04-05

**Authors:** Patrick Meister, Silvia Frisia, István Dódony, Péter Pekker, Zsombor Molnár, Stephanie Neuhuber, Susanne Gier, Ivett Kovács, Attila Demény, Mihály Pósfai

**Affiliations:** † Department of Geology, University of Vienna, Jozef-Holoubek Platz 2, 1090 Vienna, Austria; ‡ School of Environmental and Life Sciences, The University of Newcastle, Callaghan, New South Wales 2308, Australia; § Research Institute of Biomolecular and Chemical Engineering, University of Pannonia, Egyetem u. 10, 8200 Veszprém, Hungary; ∥ ELKH-PE Environmental Mineralogy Research Group, Egyetem u. 10, 8200 Veszprém, Hungary; ⊥ Institute of Applied Geology (IAG), University of Natural Resources and Life Sciences, Peter-Jordan-Straße 82, 1190 Vienna, Austria; # Institute for Geological and Geochemical Research, Research Centre for Astronomy and Earth Sciences, ELKH, Budaörsi u. 45, 1121 Budapest, Hungary; ∞ CSFK, MTA Centre of Excellence, 1121 Budapest, Hungary

## Abstract

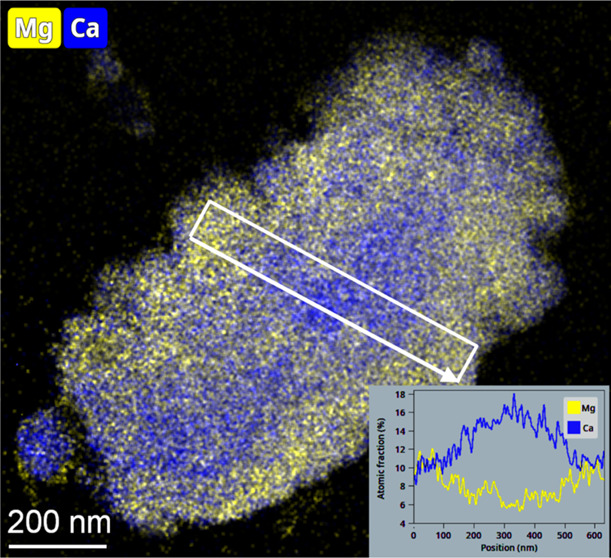

Dolomite [CaMg(CO_3_)_2_] formation under Earth surface conditions is considered largely inhibited, yet protodolomite (with a composition similar to dolomite but lacking cation ordering), and in some cases also dolomite, was documented in modern shallow marine and lacustrine, evaporative environments. Authigenic carbonate mud from Lake Neusiedl, a shallow, episodically evaporative lake in Austria consists mainly of Mg-calcite with zoning of Mg-rich and Mg-poor regions in μm-sized crystals. Within the Mg-rich regions, high-resolution transmission electron microscopy revealed < 5-nm-sized domains with dolomitic ordering, i.e., alternating lattice planes of Ca and Mg, in coherent orientation with the surrounding protodolomite. The calcite with less abundant Mg does not show such domains but is characterized by pitted surfaces and voids as a sign of dissolution. These observations suggest that protodolomite may overgrow Mg-calcite as a result of the changing chemistry of the lake water. During this process, oscillating concentrations (in particular of Mg and Ca) at the recrystallization front may have induced dissolution of Mg-calcite and growth of nanoscale domains of dolomite, which subsequently became incorporated as ordered domains in coherent orientation within less ordered regions. It is suggested that this crystallization pathway is capable of overcoming, at least at the nanoscale, the kinetic barrier to dolomite formation.

## Abstract 2

Dolomite is a sedimentary Mg−Ca carbonate mineral that forms mostly under restricted marine and evaporative lake conditions. The kinetics of dolomite formation are still not completely understood. We report the finding of sub-micrometer-scale carbonate crystals showing concentric zones of ripening to protodolomite in sediment from Lake Neusiedl, Austria. Mg-rich regions contain <5-nm-sized domains of dolomitic ordering, in crystallographic coherence with the surrounding protodolomite. This observation suggests that dolomite may form via nucleation of nanoparticles as the calcite with less Mg becomes replaced by protodolomite.

## Introduction

Dolomite is one of the most abundant carbonate minerals in the sedimentary record. However, the formation of structurally ordered CaMg(CO_3_)_2_ in both modern and ancient environments is still not completely understood. Even if many aquatic environments are supersaturated with respect to dolomite, its formation is known to be kinetically inhibited and crystallization processes are considered too slow to be directly observable under Earth surface conditions (e.g., Land).^1^ Often dolomites in the geological record show petrographic structures of pervasive, diagenetic recrystallization (e.g., Morrow; Machel; Gregg et al.).^2−4^ Yet, van Tuyl^5^ also proposed a “chemical theory” that implied that some dolomites formed while the sediment was still residing within the depositional environment. Despite its scarcity in the modern open ocean, dolomite with partial ordering occurs in several restricted marine and evaporative lake environments, in some cases as very fine-grained mud (dolomicrite; von der Borch et al.; von der Borch; Last; Teal et al.; Meister et al.; Balci et al.; McCormack et al.; Fang and Xu; Fang et al.).^6−14^ Some studies suggested, based on structural and isotopic characteristics, that dolomite may form via a spontaneous nucleation in the water column, and dolomicrite results from sedimentation after flocculation of the colloidal Ca–Mg carbonate (e.g., von der Borch et al.; von der Borch; Meister et al.; McCormack et al.; Fang et al.).^6,7,10,12,14^ These processes would be largely driven by supersaturation of the parent water with respect to a range of Mg/Ca-carbonate phases. However, step-by-step nanoscale mechanisms of dolomite precipitation, and their kinetics, remain, as yet, unsolved.

Insight into a nanoscale pathway of dolomite formation comes from high-resolution transmission electron microscopy (HR-TEM) observations of Holocene (last ca. 11,500 years) Ca–Mg carbonates. Observations of such young dolomicrites by TEM showed heterogeneous microstructures in an overall calcium-rich dolomite ascribed to the high density of defects “probably due to the growth process” (Reeder).^15^ Frisia Bruni and Wenk^16^ and Wenk et al.^17^ distinguished different types of dolomite in terms of chemical domains and microstructures in Holocene sediments. An ordered, Ca-rich dolomite with modulated microstructure was documented in association with aragonite and interpreted as replacing the orthorhombic CaCO_3_ phase. In contrast, a Mg-rich dolomite, which was not replacing a precursor aragonite and was characterized by strain contrast and “differently developed lamellar microstructures resembling agglomerates” (Wenk et al.),^17^ was interpreted as a possible “primary” precipitate, that is, implying spontaneous precipitation from a fluid. Following the idea that strain contrast would suggest primary dolomite formation, Preto et al.^18^ analyzed laminated dolomite from a Late Triassic, shallow marine evaporative setting, supersaturated with respect to dolomite. They observed dolomicrite laminae by HR-TEM and found dislocation-ridden dolomite crystals, similar to those described by Wenk et al.^17^ The HR-TEM observations of the Late Triassic dolomite revealed that micrite grains consisted of an aggregate of several tens of nanometer-sized crystals with non-uniform orientation. Preto et al.^18^ then interpreted the strong contrast and the heterogeneous microstructure of the dolomicrite as the result of lattice mismatch between single nanograins and interpreted the dolomite as being of “primary” origin.

Similar defect-ridden crystals were then reported by Lu et al.^19^ for deep-sea, hydrocarbon-seep-associated dolomites and in micritic peloidal structures within the pervasively dolomitized Late Triassic Dolomia Principale (Meister and Frisia),^20^ potentially suggesting primary precipitation of dolomicrite. In particular, the Dolomia Principale dolomicrite was interpreted as forming directly from an evaporative brine through a mechanism of poorly oriented attachment of primary dolomite nanocrystals (Meister and Frisia).^20^

Preto et al.^18^ pointed out that the concept of “primary” dolomite may not exclude its formation via a potential precursor. Indeed, using time-resolved X-ray diffraction in precipitation experiments, Rodriguez-Blanco et al.^21^ were able to observe the transition from a precursor amorphous calcium carbonate (ACC) phase to a spherulitic protodolomite, which is defined as a Mg-calcite with approximately the elemental composition of dolomite but missing the characteristic ordering peaks (Gregg et al.).^4^ While these findings provided first real-time, in situ insight into the evolution of crystallization at the sub-micrometer scale, they confirmed a classical sequence of nucleation, growth, and replacement that was reconstructed on the basis of kinetic experiments (Sibley and Bartlett; Sibley et al.; Kaczmarek and Sibley; Gregg et al.; Kaczmarek et al.).^4,22−26^ A temporal evolution, where each intermediate metastable phase forms preferentially over the more stable phase at the nanoscale, is in line with Ostwald’s step rule (Ostwald)^27^ and provides a potential route to overcome the kinetic barrier to dolomite precipitation (Liebermann; Nordeng and Sibley; Deelman).^28−31^ This was also inferred from Land’s^1^ experiments using solutions 1000-fold oversaturated with respect to the ordered CaMg(CO_3_)_2_ phase. Ripening processes may be slow and difficult to trace in natural environments, and the precursor phases are usually not observed (e.g., Tompa et al.; Nyirő-Kósa et al.; Fussmann et al.).^32−34^ However, information gained by HR-TEM investigation of dolomite from modern and ancient environments, in combination with time-resolved observations from laboratory experiments implies that these effects take place at the nanoscale. Dolomite may, thus, form through a temporal sequence of nanoscale mechanisms where kinetic barriers are subject to nanoscale interfacial energy distributions as part of a nonclassical pathway. These processes may occur while sediment grains remain in suspension. In this case, dolomite would not be diagenetic (formed post-depositionally) and its nucleation and growth would be governed by the chemical conditions prevailing in the ambient water body.

To gain further insight into nanoscale growth pathways of dolomite in modern environments, we investigated the structure and compositional domains at high resolution in single carbonate crystals in sediments cored at the bottom of a shallow (average water depth ca. 1 m), episodically evaporative, and slightly alkaline lake located in the Pannonian Basin between Austria and Hungary (Lake Neusiedl or Fertő; Figure S1; see Draganits et al.^35^ for a detailed review on the lake system). The lake sediments mostly consist of fine-grained Mg-calcite and protodolomite (e.g., Schroll and Wieden; Müller et al.; Löffler et al.).^36−38^ In general, the lake water is supersaturated with respect to several Mg/Ca-carbonate minerals due to its relatively high pH (>8.5) and high Mg/Ca ratio (>0.5 mmol/l Ca^2+^ and >4 mmol/l Mg^2+^; Fussmann et al.),^34^ but salinity, pH, and Mg/Ca ratio strongly vary between the seasons (Blohm).^39^ Near the sediment/water interface, pH is lower than in the water column above and the water is undersaturated with respect to low-Mg calcite, while remaining supersaturated with respect to the other carbonate phases, in particular protodolomite and dolomite (Fussmann et al.).^34^

Following up on the approach of Wenk et al.,^17^ we used HR-TEM, and, in addition, high-angle annular dark-field (HAADF) imaging in scanning mode (STEM) to investigate the nanometer-scale details of single grains and determine the nanostructures and their crystallographic orientations. The nanostructural analyses were complemented with elemental maps recorded by energy-dispersive X-ray spectroscopy (EDX) in STEM mode. We then evaluated and reconstructed potential nanoscale pathways for “primary” dolomite formation in a natural depositional environment.

## Results and Discussion

### Compositional and Mineralogical Variety of Authigenic Mg–Ca Carbonates

**Figure 1:**
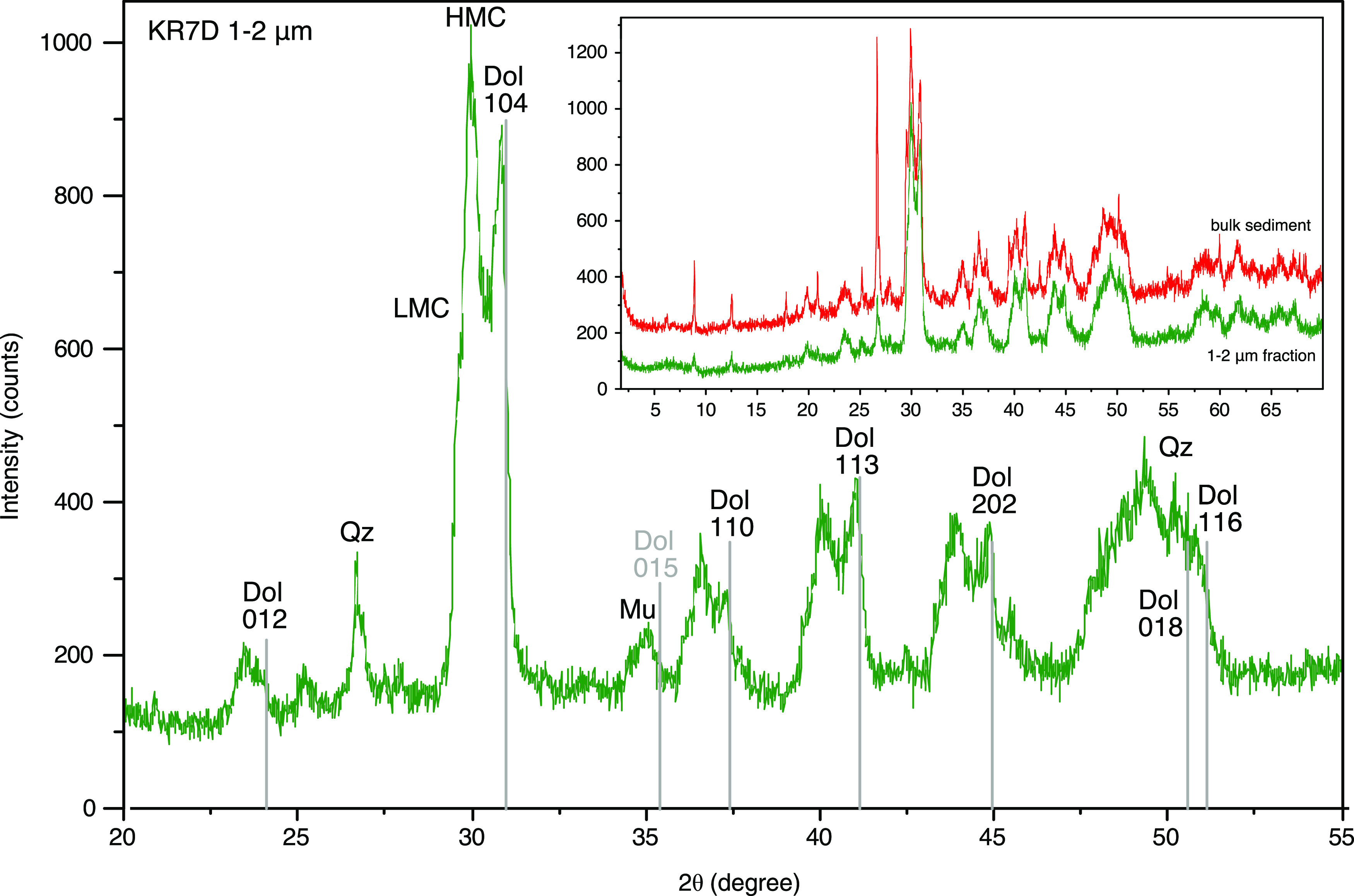
XRD pattern of grain-size fraction 1–2 μm of sample KR7D-2. The spectrum reveals carbonate phases ranging from low-Mg calcite to protodolomite, with three peaks standing out. The presence of the dolomite 015 peak (indicating Ca/Mg ordering) cannot be detected. The inset shows that the grain size separate is representative of the phases present in the bulk sample. LMC, low-Mg calcite; HMC, high-Mg calcite; Dol, dolomite; Qz, quartz; Mu, muscovite.

Sediment cored at Lake Neusiedl largely consists of fine-grained mud (Figure S2). X-ray diffraction (XRD) suggests that carbonate phases ranging in composition (and cell parameters) between low-Mg calcite and protodolomite, with three peaks standing out (Figure 1), are intermixed with clay minerals and quartz. Fourier transform infrared (FTIR) spectra analyses were conducted on untreated samples as well as on samples freeze-dried for 8 h. No change was observed except the water content decrease that resulted in lower peaks at about 3400 cm^–1^. Figure S3 shows the results of freeze-dried samples. None of the samples indicated the presence of amorphous calcium carbonate (ACC), whose typical spectrum is also shown for comparison (after Demény et al.).^40^ Larger mud clasts in the 100-μm-size range are aggregates of colloidal particles (Figure S4), similar to those documented in Late Triassic peritidal facies (Meister and Frisia),^20^ although they are not cemented by subsequent postdepositional processes.

Analysis of the 1–2 μm fraction by STEM combined with EDX revealed up to 1-μm-scale rhombohedral crystals, showing somewhat subhedral shapes, and polycrystalline aggregates (Figure S5). Overall, Mg/Ca ratios as determined from STEM-EDX elemental maps show an almost continuous distribution from low-Mg calcite to stoichiometric dolomite (see the histogram in Figure S5). Based on the rather diverse population of Mg–Ca carbonate grains with variable compositions, the carbonates are considered to be largely authigenic (Neuhuber et al.).^41^

#### Processes Leading to Compositional Variation of Mg–Ca Carbonate Crystals

**Figure 2:**
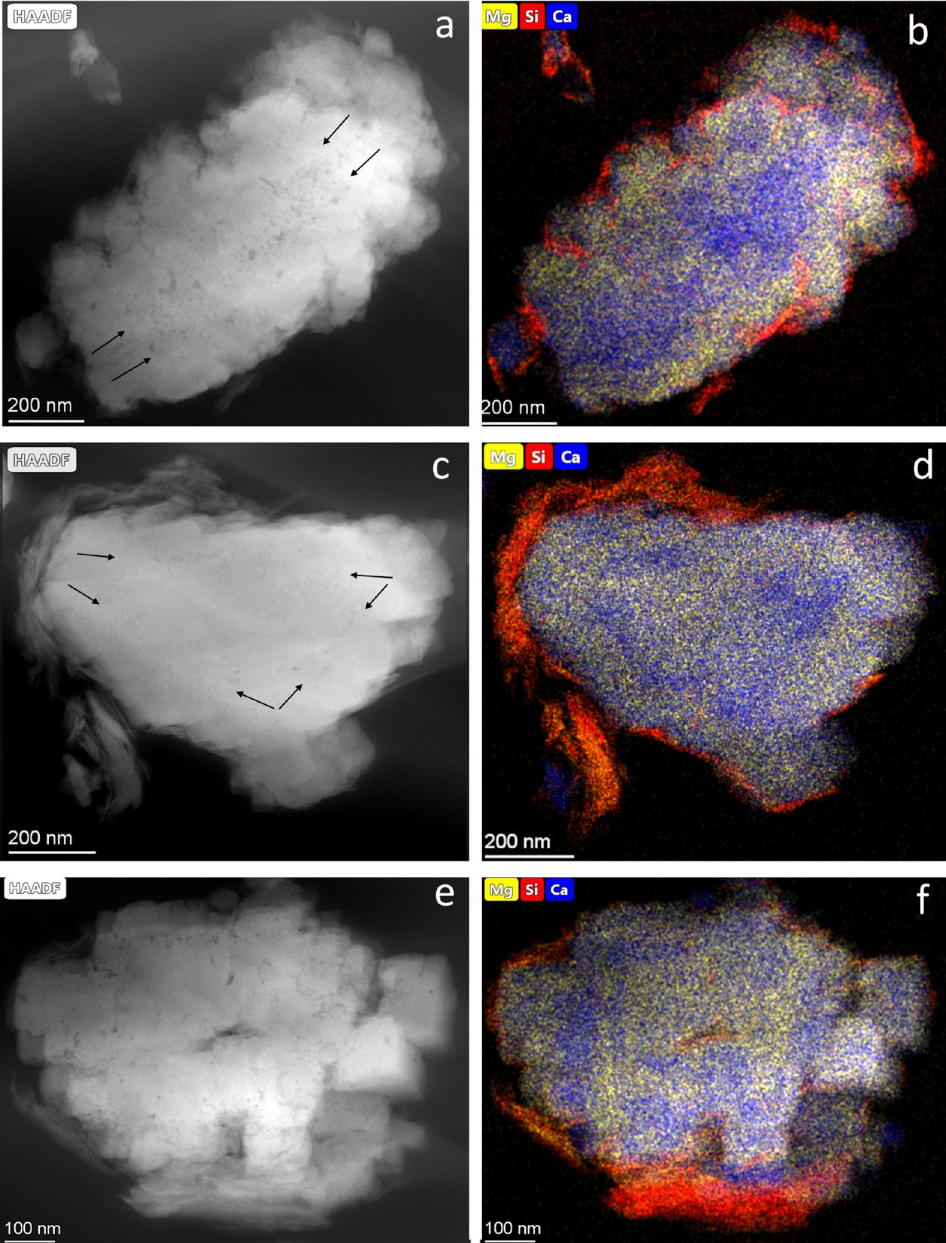
Pairs of HAADF images and elemental maps showing the heterogeneous distributions of Ca, Mg, and Si in three carbonate particles (all from an ion-milled sample; supposedly their thicknesses are uniform and thus variation in thickness contributes negligibly to the HAADF contrast). In the HAADF images, swarms of small voids appear as spots with dark contrast. Zones with numerous voids are marked by arrows in (a) and (c). High Si concentrations indicate flakes of the clay mineral smectite, both surrounding the carbonate particles and being incorporated in them.

Some grains show a concentric zonation with enrichment of Mg at the rims (Figure 2), while others show zonation in the reverse order, with Mg enrichment at the center. Based on selected-area electron diffraction (SAED) patterns (Figure S6), the grains show a coherent crystal lattice across different compositional domains. Strongly pitted and partially dissolved areas are clearly visible (Figure 2), and high number of voids mostly coincide with Mg-poor zones (with Mg/Ca mol ratio < ∼0.5).

The Mg-poor and Mg-rich zones mark a compositional variation reflecting changing water chemistry in response to the balance between recharge and evaporation. Concentric zoning may result from these varying conditions during growth, but may also be due to a replacement front that migrates from the margin toward the center of the crystal. Carbonate grains showing different zonation may, in fact, be a consequence of the ripening of Mg-calcite^42^ into protodolomite within the boundary layer between the sediment and the water column, during times when the shallow lake water is characterized by low supersaturation (Fussmann et al.).^34^ The fact that some crystals show an inverse zonation, with Mg-rich composition at the center, potentially documents a complex formation history, with different generations of crystals experiencing different water chemistry conditions in different growth phases.

### Dolomitic Nanodomains

**Figure 3:**
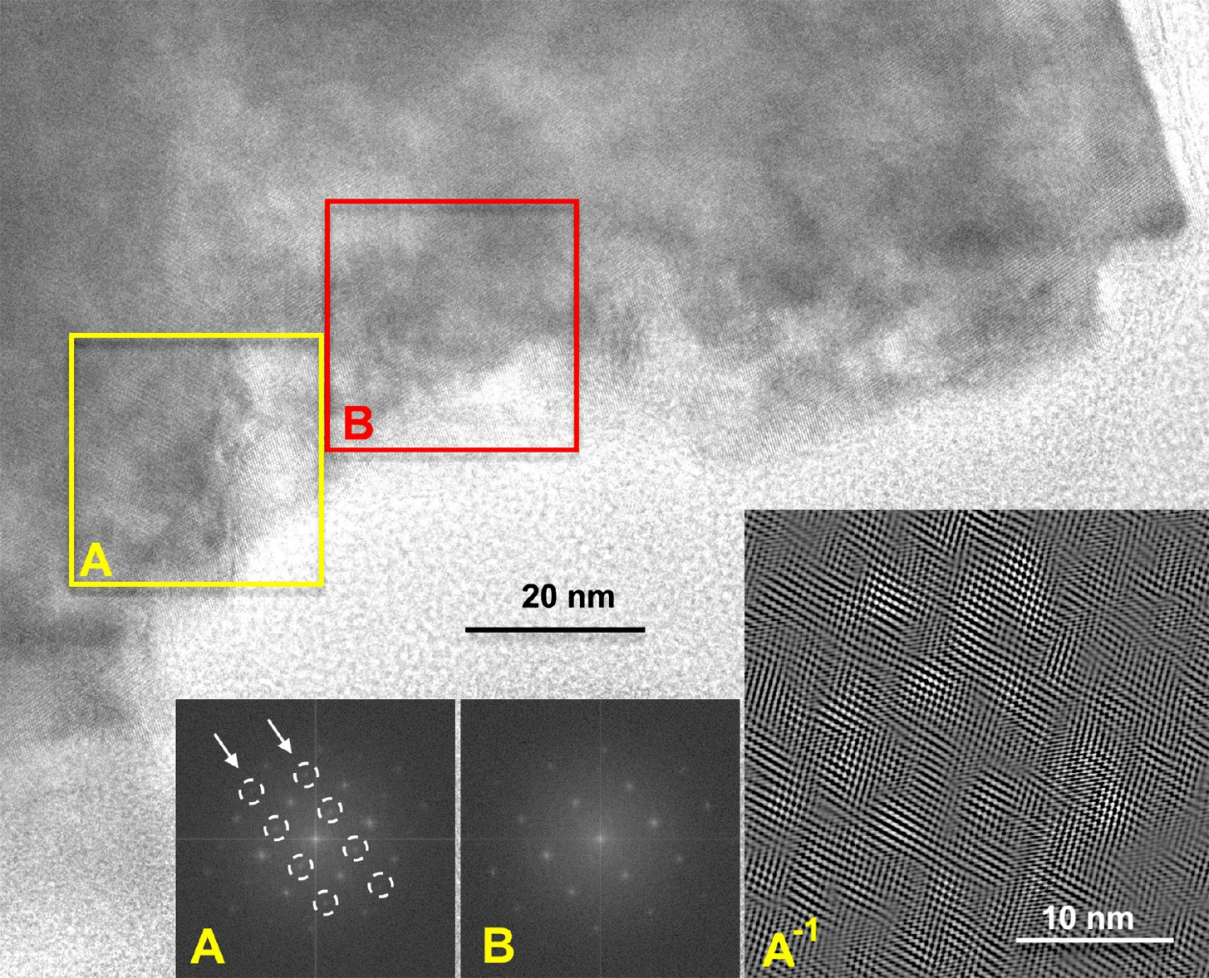
High-resolution transmission electron microscopy (HRTEM) image and FFTs of the boxed areas marked A and B, and an inverse FFT of A (the inset in the bottom right, at higher magnification), made using only the circled spots in A. While the FFT from B does not show ordering reflections (*b*-reflections), the one from A does (in the arrowed rows). The inverse FFT displays (in bright contrast) the regions that produced the extra (circled) reflections.

HR-TEM images reveal crystals with coherent crystallographic orientation in entire grains; according to the SAED patterns, the different concentric compositional zones are also coherent in orientation. However, the image contrast varies between the grains (Figure 3). In places, fast Fourier transform (FFT) patterns of HR-TEM images from selected domains show additional rows of reflections that are identified as *b*-type dolomite reflections (Reeder and Nakajima;^43^ marked by dashed circles in Figure 3A), whereas other domains in the same grain lack such reflections, suggesting calcite structure (Figure 3B). If the inverse FFT image is generated by using only the *b*-type dolomite reflections within area A, domains of ca. 2 nm in size become apparent (Figure 3, bottom right inset).

**Figure 4:**
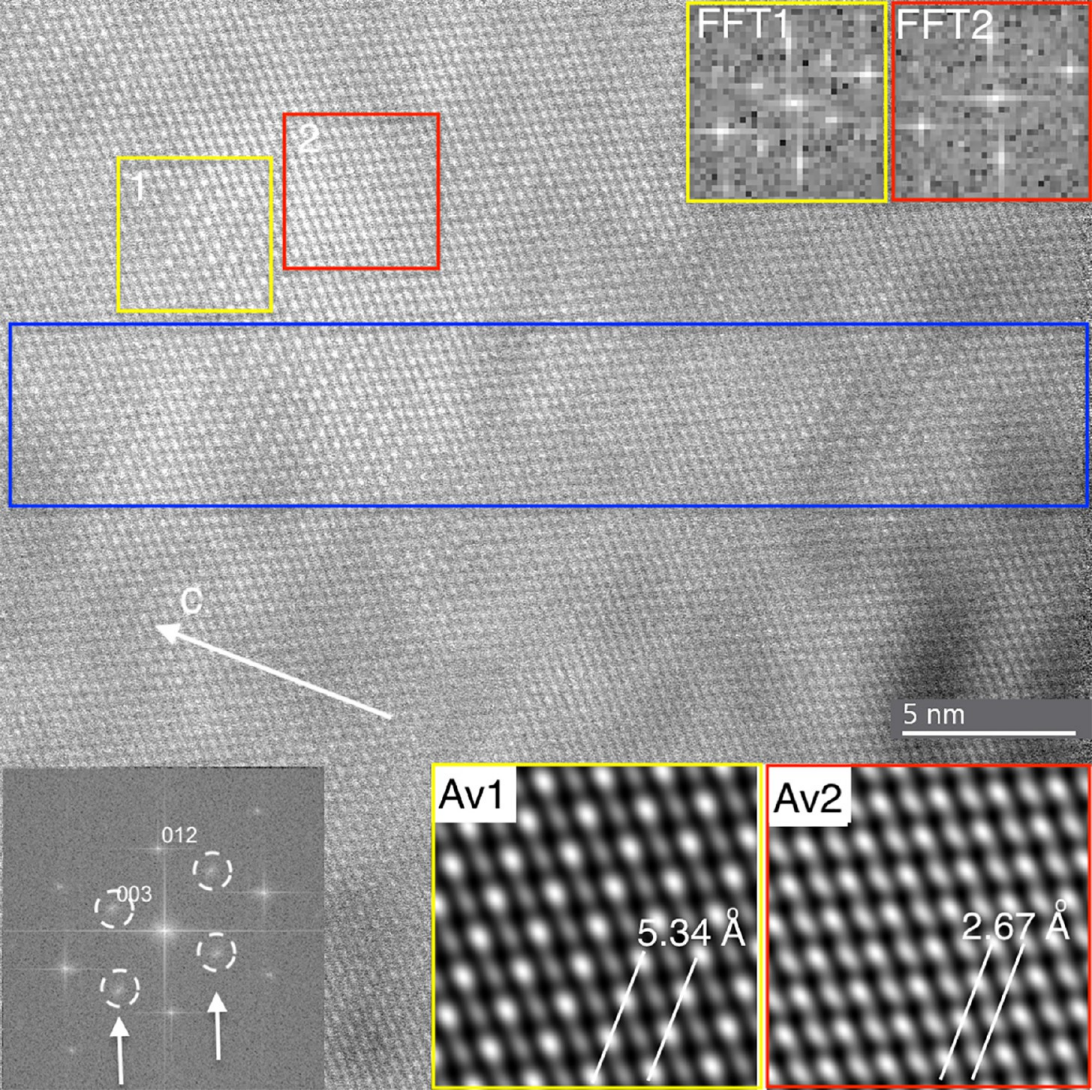
High-resolution HAADF image of a region of a carbonate grain with a dolomitic composition (Ca/Mg = 1). Patches with variable contrast (such as the ones marked 1 and 2) can be observed, but most of the image shows double periodicity along *c* (with respect to calcite), as indicated by the inserted FFT calculated for the entire area (in the bottom left), in which the white arrows mark rows of *b*-reflections that would be absent in calcite. The yellow and red boxes mark areas with full and no ordering of Ca and Mg, respectively; their FFTs are shown in the top right (FFT1 and FFT2), and lattice-averaged images in the bottom right (Av1 and Av2). The blue box indicates the region shown in Figure 5.

**Figure 5:**
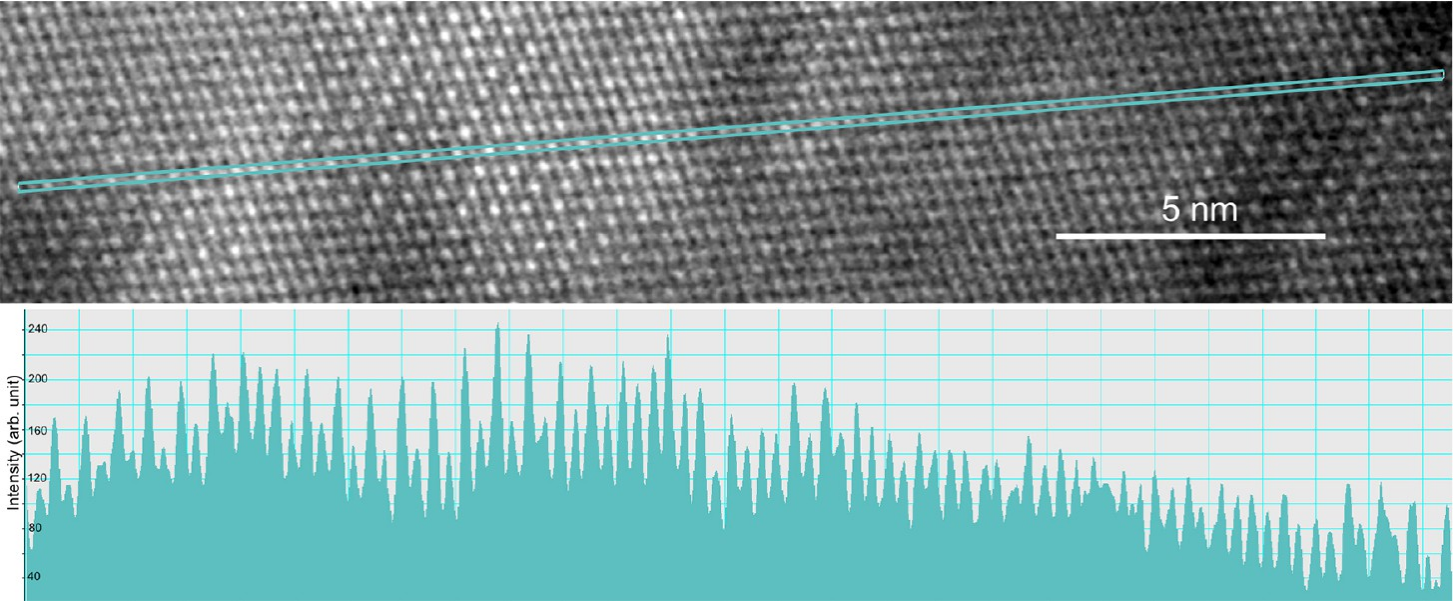
High-resolution HAADF image (the area within the blue box marked in Figure 4) and an intensity profile along the indicated row of spots. While in certain segments more and less intense spots alternate, in other segments along the profile, the intensities of successive spots are nearly equal, suggesting that more and less ordered patches occur, without clear boundaries between them.

Results from HAADF images show nanometer-scale domains with either calcite or dolomite structure (Figure 4), as indicated by both the changes in observable periodicities (single or double, as in calcite and dolomite, respectively) along the *c* axis and the distinct FTs of the two regions. In HAADF images, the intensity in each pixel is directly related to the square of the atomic number, provided that the composition and the thickness of the particle are uniform. Thus, the brightness of an individual spot representing a cation column is related to its composition, i.e., Mg/Ca ratio. The Mg/Ca ratios within individual atomic columns were determined from the image contrast in both ordered and disordered regions. Within the ordered regions, such as the one marked by the yellow box in Figure 4, the intensity ratios of alternating atomic columns represented by bright vs. muted spots suggest the ordering of Ca and Mg. In places within the ordered (dolomitic) domains, adjacent cation columns can be characterized by average compositions of nearly pure Ca and Mg, whereas the apparently disordered (calcite-like) regions (such as that marked by the red box in Figure 4) can be characterized by adjacent cation columns with approximately identical Mg/Ca ratios. Longer segments of intensity profiles along distinct rows of spots show great variations in the degree of ordering. An example is shown in Figure 5, where a sharp transition between the ordering domains is not clearly observable.

#### Potential Pathway of Nanodomain Formation

Ordering domains similar to those observed in Mg/Ca carbonate from Lake Neusiedl have been described by Fang and Xu^13,44^ in both Ordovician, marine dolomites and less than 2000 years old dolomites formed in a saline lake. HAADF patterns resolve the alternation of Mg-rich and Mg-poor lattice planes within the ordered domains, confirming that a cation ordering occurs, giving rise to a dolomitic structure showing *b*-reflections (Figures 3 and 4). While the precise pathway of formation of the observed nanoscale domains has not been clarified, there are several processes that could give rise to such structures.

In a classical growth pathway via attachment of monomers to a growing crystal surface, a distribution of relatively distinct domains could be explained with oscillating conditions within the surface boundary layer of the fluid, as it has been inferred as causing Liesegang banding (e.g., Barge et al.).^45^ The compositional variations would induce alternating thermodynamic and kinetic conditions, which would result in contrasting mineralogical structures, while the lattice still forms in a coherent way.

Such oscillating conditions could also occur in the relatively small interstitial gap at a recrystallization front, in the fluid between a dissolving and a precipitating phase, giving rise to oscillatory composition in the overgrowing crystals (Merino; Ruiz-Agudo et al.).^46,47^ Frisia^48^ documented the occurrence of microporosity at the boundary between a Ca-rich dolomite and a more stable, stoichiometric dolomite that replaced the calcian dolomite, as well as between dolomite and an aragonite precursor. It would then stand to reason that the nanoporosity documented here, in modern and natural Ca–Mg carbonates (see STEM images in Figure 2) could reflect localized dissolution and reprecipitation. It is also possible that the nanoporosity inherited from a hydrated, amorphous calcium carbonate or Ca/Mg-carbonate precursor (e.g., Goodwin et al.)^49^ could have contributed to intracrystalline permeability. Renard et al.^50^ suggested that natural crystals may generally be porous and that pores provide conduits for fluid to reach the crystallization front through the interior of the crystal. Depending on the pore size and possibly limited connectivity, intracrystalline porosity most likely imposes diffusion limitation. Furthermore, smectite-group clay minerals that are tightly associated with the carbonates (Figure 2) may affect permeability and thereby contribute to diffusion-limited conditions (Molnár et al.).^51^ Given the dynamics of dissolution and precipitation, in combination with diffusion limitation, this could have caused local variation in solution composition and supersaturation. The domains then would have formed as a result of auto-induced modulation of solution composition at the growth front, which could occur due to dynamics of diffusion vs dissolution/precipitation (see Barge et al.).^45^ Putnis et al.^52^ and Putnis and Ruiz-Agudo^53^ show by atomic force microscopy (AFM) that novel exotic phases occur as a result of local supersaturation, and local conditions may also explain the formation of dolomitic domains.

Alternatively, nanodomains could have been inherited from a precursor that already contained inclusions of a phase embedded in a surrounding matrix of a different phase, e.g., nanoparticles that already contained a motif of a dolomitic structure within an amorphous matrix. This would then require a fabric retentive replacement, in which the structural information is transferred across the fluid gap at the recrystallization front (topotactic effect). However, experiments such as the ones shown by Rodriguez-Blanco et al.^21^ show no preservation of morphologies of an amorphous calcium carbonate precursor, and no amorphous precursor was detected in the sediment from the lake. Also, calcite with a lower Mg content as potential precursor for protodolomite observed in the present study shows no signs of nanodomains, such as the dolomitic domains described above. More likely, thus, the ordered nanodomains have truly resulted from nucleation at the recrystallization front.

#### Domain Formation via Oriented Attachment and Incorporation of Nanocrystals

According to the classical nucleation theory, particles below the critical size would be unstable and, hence, unlikely persist within a solution. Nevertheless, it has been shown that nanoscale particles could exist in a metastable state (Baumgartner et al.).^54^ As the capillary assumption (i.e., flat surfaces) cannot be upheld for such small particles, their energetic state is difficult to describe by classical theories. Verch et al.^55^ and Gebauer et al.^56^ suggested that nanometer-size calcium–magnesium pre-nucleation-clusters (molecular clusters, PNCs) exist, which may be considered as species that persist in solution according to the mass action law. De Yoreo et al.^57^ proposed that a large diversity of nanoparticles, ranging from amorphous, molecular clusters to nanocrystals with a well-ordered crystal structure may exist due to energy minima of a complex nanoscale energy landscape. Nanoparticles would, thus, be independent of the stability of the corresponding macroscopic phases. They would be expected to form with the competitive sequence of metastable states with increasing energy barriers, thus conforming to Ostwald’s step rule (Ostwald;^27^ Cölfen and Mann,^58^ Figure 1 therein), and this sequence would ultimately control the pathway of crystal formation. In the case of dolomite, it is conceivable that the nanoscale energetic state of the nanoparticles could be decisive to overcome (or by-pass) the large kinetic barrier to dolomite formation.

Indeed, the ordered nanodomains in Mg–Ca carbonates from Lake Neusiedl comprise a few atomic layers, in the size range of some of the nanoparticles discussed above, some of which may already carry a dolomite structure. To form a coherent lattice, however, the nanocrystals would have to attach in a perfectly oriented way. That nanocrystals of this size can attach in an oriented way has been demonstrated for iron oxides, using liquid phase, in situ HRTEM (Li et al.; Zhu et al.).^59,60^ The nanocrystals reaching the surface of a growing macroscopic crystal have a round to irregular shape, and they rotate until they find the right lattice orientation, then immediately form bonds with a few atoms at the surface. The gaps are then filled by mobilizing some monomers from the nanocrystal surface or from the solution, thereby reducing the Gibbs free energy of curvature.

**Figure 6:**
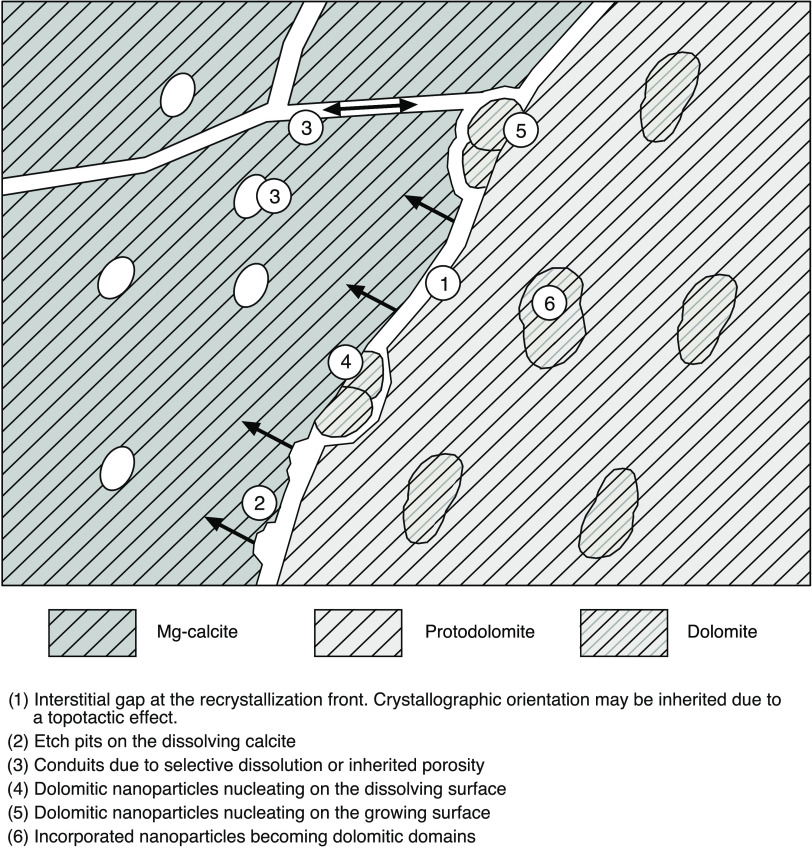
Graphical overview of potential pathways leading to nanoscale ordered dolomitic domains in crystallographic coherence with surrounding Mg-calcite. A metastable, precursor phase (Mg-calcite) precipitates from the solution and is replaced by more stable phases (protodolomite) along a recrystallization front. The precursor is dissolved by circulating fluids along an interstitial gap, where ions can be removed and delivered via intracrystalline porosity. Due to local fluctuation of ionic composition, nanoparticles are deposited at either the dissolving or the growing crystal surface. The nanoparticles may nucleate in crystallographic continuity with the existing crystal structures. As a further step, the nanocrystals become incorporated in the growing, secondary phase.

Recently, it has been found that this process also occurs in carbonates (Gehrke et al.; Liu et al.; Takasaki et al.; Sun et al.).^61−64^ Observations by AFM on dissolving mineral surfaces by Putnis et al.^52^ and Putnis and Ruiz-Agudo^53^ demonstrated that nanoparticles also form during dissolution of diverse minerals. For example, Mg-carbonate nanoparticles form on the surface of brucite, which is dissolving and forming etch pits (Figure 4 in Putnis and Ruiz-Agudo).^53^ The nanocrystalline phase could in this way grow in crystallographic continuity with another secondary phase, in which it would become embedded as nanodomains (Figure 6). Local conditions may exist at the dissolving mineral surface that stabilize particular phases at the nanoscale. And these local conditions may result from the dynamics of dissolution and reprecipitation at a recrystallization front (Figure 6). In this way, a nano-particulate pathway may play a role during ripening processes under variable conditions in the parent water.

Alternatively, nanodomains could form as a result of spinodal decomposition of the fluid (e.g., Seknazi et al.; Bianco-Stein et al.).^65,66^ However, spinodal decomposition would occur at high supersaturation and would, thus, lead to the formation of a metastable phase, rather than the ordered phase. Hence, a scenario of nucleation of ordered nanoparticles at the recrystallization front would better explain the finding of dolomitic nanodomains.

#### Overcoming the Kinetic Barrier to Nano-Dolomite Formation

How the above-mentioned oscillations in interstitial solution composition could result in dolomitic ordering still remains to be explained. Dolomite ripening under oscillating conditions, in accordance with Ostwald’s step rule, was already postulated by Lieberman,^28^ Nordeng and Sibley,^29^ and Deelman.^31^ To understand how oscillations can help to overcome the kinetic barrier to ordered dolomite, one needs to understand the nature of this barrier. Several concepts propose that the dehydration of Mg ions represents the major energy barrier inhibiting dolomite formation (e.g., Petrash et al.^67^ and references therein). This was already proposed by Lippmann,^68^ but it does not explain why protodolomite readily forms, while ordered dolomite remains inhibited. Recent experiments have demonstrated that the kinetic barrier still exists in a water-free solvent (Xu et al.).^69^ Instead, it is to be expected that the kinetic barrier is specific for the mineral, in this case for cation ordering. Energy has to be invested to reduce entropy by ordering cations in alternating lattice plains so that the energy barrier of dolomite formation is most likely of entropic nature.

The main energy barrier for dolomite formation is most likely related to the mineral surface, particularly to the interfacial energy of a growing particle. The interfacial energy would be substantially affected under oscillating environmental conditions. Moreover, nanoscale effects modify interfacial energy at a nanoscale interfacial energy landscape as outlined by De Yoreo et al.^57^ The exact nature of this energy landscape cannot be resolved at present by using solely macroscopic theory, but future developments of energetic modeling at the molecular scale may offer a potential route to capture and predict the energetic (and entropic) barrier for nanoscale dolomitic ordering.

### Long-Term Ripening of Mg/Ca-Carbonates

The pathway of nucleation along the recrystallization front outlined above may explain the formation of ordered nanodomains in crystallographic continuity with the surrounding phase. Nevertheless, this assemblage of metastable protodolomite with dolomitic nanodomains still represents a transient one, which is not commonly preserved in the geological record. The transition may proceed slowly, as suggested by the relatively high ^14^C age (for some carbonate fractions > 1 ka; Neuhuber et al.^41^). However, Fussmann et al.^34^ highlighted the importance of variation in hydrochemical composition for ripening of the metastable to a stable phase in accordance with Ostwald’s step rule. In this sequence, a metastable, perhaps amorphous, precursor may have formed first via spontaneous nucleation. Lower saturation near the sediment/water interface, due to lowered pH, may have then induced recrystallization and ripening of any potential precursors. As this process occurs in contact with the surface lake water, ripening would not be considered diagenetic, that is, not “postdepositional”.

Critically, in the Holocene lake sediment retrieved downcore, even the underlying laminated layers (that could not have been involved in the permanent resuspension and mixing) did not show a fundamental shift in mineralogic composition. It appears as if the mineral assemblage has not changed much during the entire deposition history during which several decimeters of sediment have accumulated, and ripening may not have occurred at a continuous rate. While the metastable phase is residing in contact with pore fluid under permanent supersaturation (with respect to the metastable phase), it could not have dissolved and, thus, not recrystallized to the stable phase (Fussmann et al.).^34^ Only upon changes in the porewater composition, leading to undersaturation of the fluid with respect to the metastable phase, may ripening to the stable phase proceed, as observed in modern dolomites in several cases (e.g., McKenzie; Gregg et al.).^70,71^ This could be induced by a larger-scale environmental change or over geological time scales, if a sedimentary unit gets buried and decoupled from the surface water system. This process may also be supported by the existence of primary or secondary nanoporosity providing nanoscale conduits within the grains, driving pervasive dolomitization (e.g., Olanipekun and Azmy).^72^

## Conclusions

Ongoing dolomite formation in a shallow, slightly alkaline lake (Lake Neusiedl) involves a nanoscale, nonclassical growth pathway, which is part of an evolutionary sequence proceeding episodically, in response to changes in the surrounding aqueous chemistry. High supersaturation leads to rapid nucleation of Mg calcite, which recrystallizes to protodolomite at lower supersaturation conditions. Oscillation in chemical composition may occur as a result of dissolution/reprecipitation at the recrystallization front, giving rise to local supersaturation relative to dolomite and nucleation of nanoparticles showing dolomitic structural ordering. The nanoparticles are incorporated in crystallographic continuity within a secondary Mg-calcite phase, in which they appear as nanodomains.

The transformation of the heterogeneous Ca–Mg carbonate into ordered dolomite may be facilitated by nanoporosity, providing conduits for diagenetic transformation. Oscillating conditions at the nanoscale could not only cause selective supersaturation of the stable phase but affect the nonclassical surface energy landscape, lowering a potential entropic barrier for the ordering of cation layers in incipient domains of ordered dolomite. The finding of both nanoscale ordered dolomitic domains within disordered Ca–Mg phases and nanoporosity, which may favor dissolution-reprecipitation, provides a piece of the puzzle of dolomite formation.

## Material and Methods

The site where sample KR7D was collected is located within the lake (N 47°48′53.68″, E 016°42′19.42″), and the sample was retrieved by boat using an acrylic glass corer that was pushed into the sediment. The top 25 cm of the sediment was homogeneous, very fine-grained, dark gray, and water-saturated. The lower part between 25 and 50 cm was different and consisted mostly of clay with a lower water content and, thus, showed a more adhesive consistency. The water-saturated sediment slice (volume of ca. 750 mL) at the top of each core was separated using a spatula and sealed in plastic bags for further examination.

The grain size fraction 1–2 μm was separated from the sediment by centrifugation and mineralogically characterized with powder X-ray diffraction (XRD) (Panalytical PW 3040/60 X’Pert PRO, Cu Kα radiation, 40 kV, 40 mA, step size 0.0167° 2θ, 5 s per step) at the Department of Geology, University of Vienna.

Bulk sediment was embedded in LR-white resin and subsequently polished with SiC powder (0.25 μm grain size), then with Logitech Syton SF1 colloidal silica (pH 10; 0.032 μm grain size; Logitech Ltd., Old Kilpatrick, Glasgow, G60 5EU, U.K.). Micro-XRD was performed at the Hungarian Academy of Sciences, Budapest. The RIGAKU D/MAX RAPID II diffractometer is a combination of a MicroMax-003 third-generation microfocus, sealed tube X-ray generator, and a curved imaging plate detector. The diffractometer is operated with Cu Kα radiation generated at 50 kV and 0.6 mA. The IP was read by a laser scanning readout system in approximately 1 min. 2DP RIGAKU software was used to record the diffraction image from the laser readout, allowing the operator to determine the area to integrate for a 2θ vs intensity plot. This plot was read into the RIGAKU PDXL 1.8 software for data interpretation.

Attenuated total reflection (ATR) Fourier transform infrared spectroscopy analyses were performed using a Bruker Vertex 70 spectroscope at IGGR-RCAES, Budapest. For each sample, 32 scans were recorded in the 4000–400 cm^–1^ spectral range, with a resolution of 2 cm^–1^. Approximately 3 mg samples were measured three times. The data were processed with the OPUS 8.1 software.

Samples for transmission electron microscopy (TEM) were prepared by depositing a drop of aqueous suspension of sediment particles on copper TEM grids covered by an ultrathin amorphous carbon film, as well as by embedding sediment particles in a resin and thinning them to electron-beam transparency using an argon ion milling system. TEM analyses were performed using a Talos F200X G2 instrument (Thermo Fisher), operated at a 200 kV accelerating voltage, equipped with a field emission gun and a four-detector Super-X energy-dispersive X-ray spectrometer, and capable of working in both conventional TEM and scanning transmission (STEM) modes. Low-magnification bright-field (BF) images, high-resolution (HRTEM) images, and selected-area electron diffraction (SAED) patterns were obtained in TEM mode. STEM high-angle annular dark-field (HAADF) images were collected both for high-resolution structure analyses and for mapping elemental compositions by coupling STEM imaging with energy-dispersive X-ray spectrometry (EDX).

## Supporting Information Available

Map showing the location of Lake Neusiedl and the sampling site (Figure S1); SEM images of Mg-calcite and protodolomite (Figure S2); Fourier transform infrared spectra of the studied samples as well as a typical spectrum for amorphous calcium carbonate (Figure S3); micro-XRD diagram of embedded bulk sediment (Figure S4); elemental map and histogram (Figure S5); and example of SAED patterns obtained from Mg-rich parts of carbonate grain (Figure S6) (PDF)
