# Supplementary material for: Nanoscale Pathway of Modern Dolomite Formation in a Shallow, Alkaline Lake
Source: Cryst Growth Des. 2023 Apr 5;23(5):3202–12. doi: 10.1021/acs.cgd.2c01393 (PMC10162443; doi:10.1021/acs.cgd.2c01393)
Supplement: Supplementary file 1 — cg2c01393_si_001.pdf [file cg2c01393_si_001.pdf]

Supplementary material to:

Nanoscale pathway of modern dolomite formation in a shallow, alkaline lake

Patrick Meister[1], Silvia Frisia[2], István Dódony[3], Péter Pekker[3], Zsombor Molnár[3,4], Stephanie Neuhuber[5], Susanne Gier[1], Ivett Kovács[6,7], Attila Demény[6,7], and Mihály Pósfai[3,4]

[1] Department of Geology, University of Vienna, Josef-Holaubek Platz 2, 1090 Vienna, Austria
[2] School of Environmental and Life Sciences, The University of Newcastle, Callaghan, New South Wales, 2308, Australia
[3] University of Pannonia, Research Institute of Biomolecular and Chemical Engineering, Egyetem u. 10, 8200 Veszprém, Hungary
[4] ELKH-PE Environmental Mineralogy Research Group, Egyetem u. 10, 8200 Veszprém, Hungary
[5] Institute of Applied Geology (IAG), University of Natural Resources and Life Sciences, Peter-Jordan-Straße 82, 1190 Vienna, Austria
[6] Institute for Geological and Geochemical Research, Research Centre for Astronomy and Earth Sciences, ELKH, Budaörsi út 45, 1112 Budapest, Hungary
[7] CSFK, MTA Centre of Excellence, Konkoly Thege Miklós út 15-17, 1121 Budapest, Hungary

As supporting information, we provide additional figures cited in the main text. The location of sampling is indicated on the map in Fig. S1. While the sediment has been described in detail in Fussmann et al. (2020), we provide additional analytical results. For the method descriptions see the main text.

Mineralogical analyses

Scanning electron microscope (SEM) images show crystals of Mg-calcite (Fig. S2A) with an aggregate-looking morphology (but according to TEM coherent lattice) and protodolomite (Fig. S2B) with a well-defined rhombohedral shape. Fourier transform infrared spectrometry (FTIR; Fig. S3) confirms the presence of calcite and dolomite, but absence of any potential amorphous precursor phases.

Micro-X-ray diffraction patterns of untreated sediment (Fig. S4) show the larger scale distribution of minerals in the sediment: a dolomitic mud clast is embedded within homogeneous mud, composed predominantly of Mg-calcite.

Element distribution

Energy-dispersive X-ray (EDX) analysis performed in scanning transmission electron microscope (STEM) mode (Fig. S5A) reveals that distribution of aggregate-like, multifaceted grains of Mg-calcite showing nevertheless a coherent crystal lattice and well-defined rhombohedra showing a more uniform elemental composition. The histogram in Fig. S5B confirms that no distinct phases dominate but that almost the entire spectrum of Mg/Ca ratios is represented between the compositions of calcite and dolomite.

Nano-scale dolomitic domains

To further characterize the dolomitic nano-domains, selected-area electron diffraction (SAED) patterns are compared for a dolomitic domain (Fig. S6A) with the surrounding Mg-calcite (Fig. S6B). The diffraction pattern for the dolomitic domain shows a higher degree of cation ordering (as suggested by the strong intensity of *b*-type reflections, in the rows marked by red arrows) than the one for the protodolomite part (that shows barely any intensity at the positions of b-type reflections).

References

Fussmann, D.; von Hoyningen-Huene, A. J. E.; Reimer, A.; Schneider, D.; Arp, G.; Daniel, R.; Babková, H.; Peticzka, R.; Maier, A.; Meister, P. Authigenic formation of Mg-Ca-carbonates in shallow alkaline water in Lake Neusiedl, Austria. *Biogeosciences* **2020**, *17*, 2085-2106. https://doi.org/10.5194/bg-2019-449

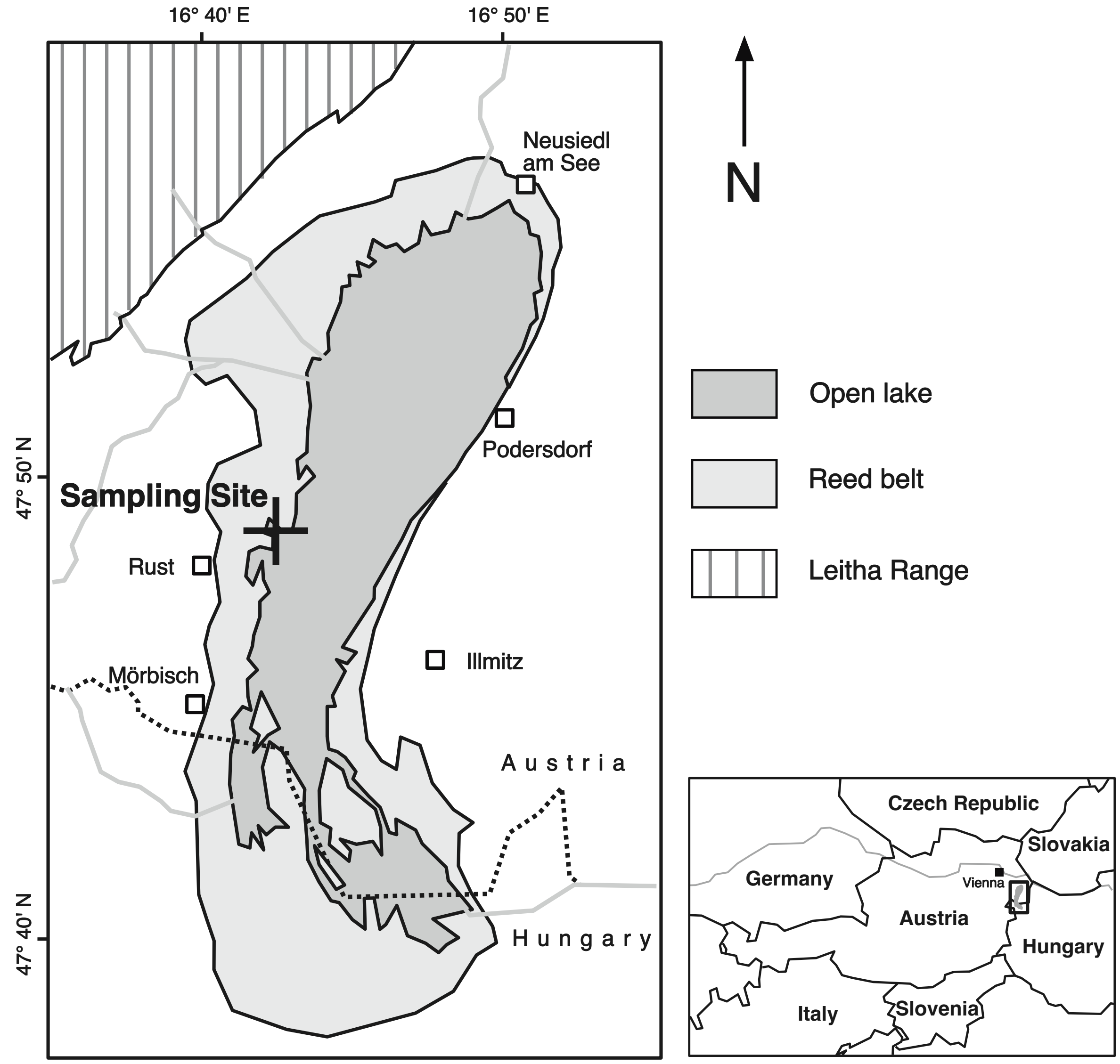

Fig. S1. Map showing the location of Lake Neusiedl and the sampling site.

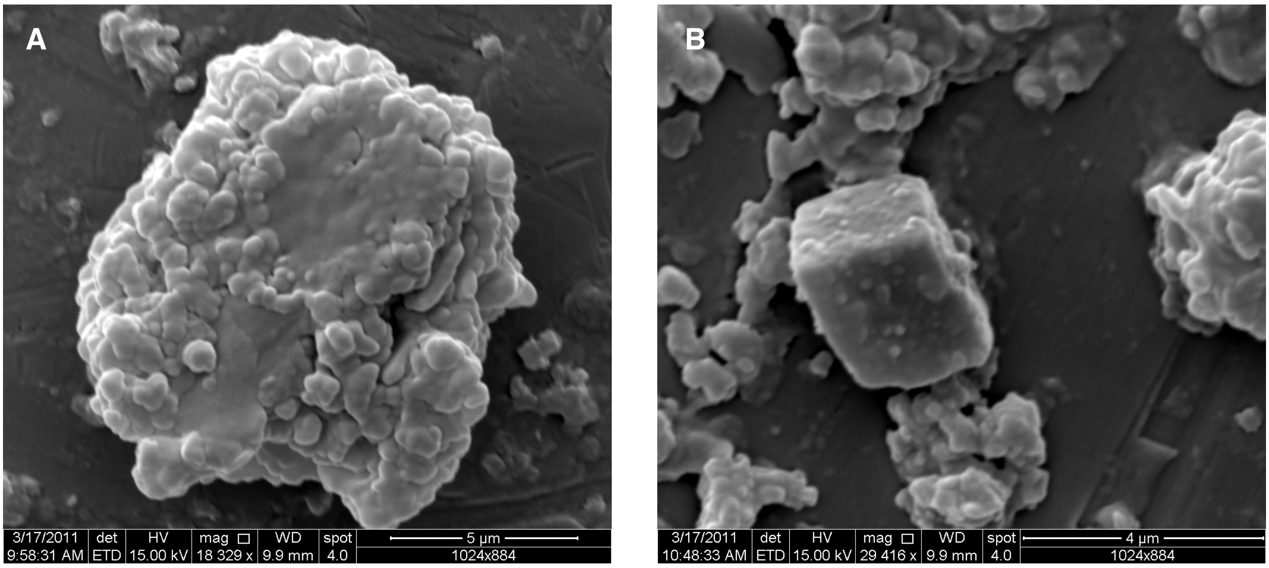

Fig. S2. Scanning electron microscope images of (A) Mg-calcite and (B) protodolomite, intermixed with clay minerals and detrital quartz.

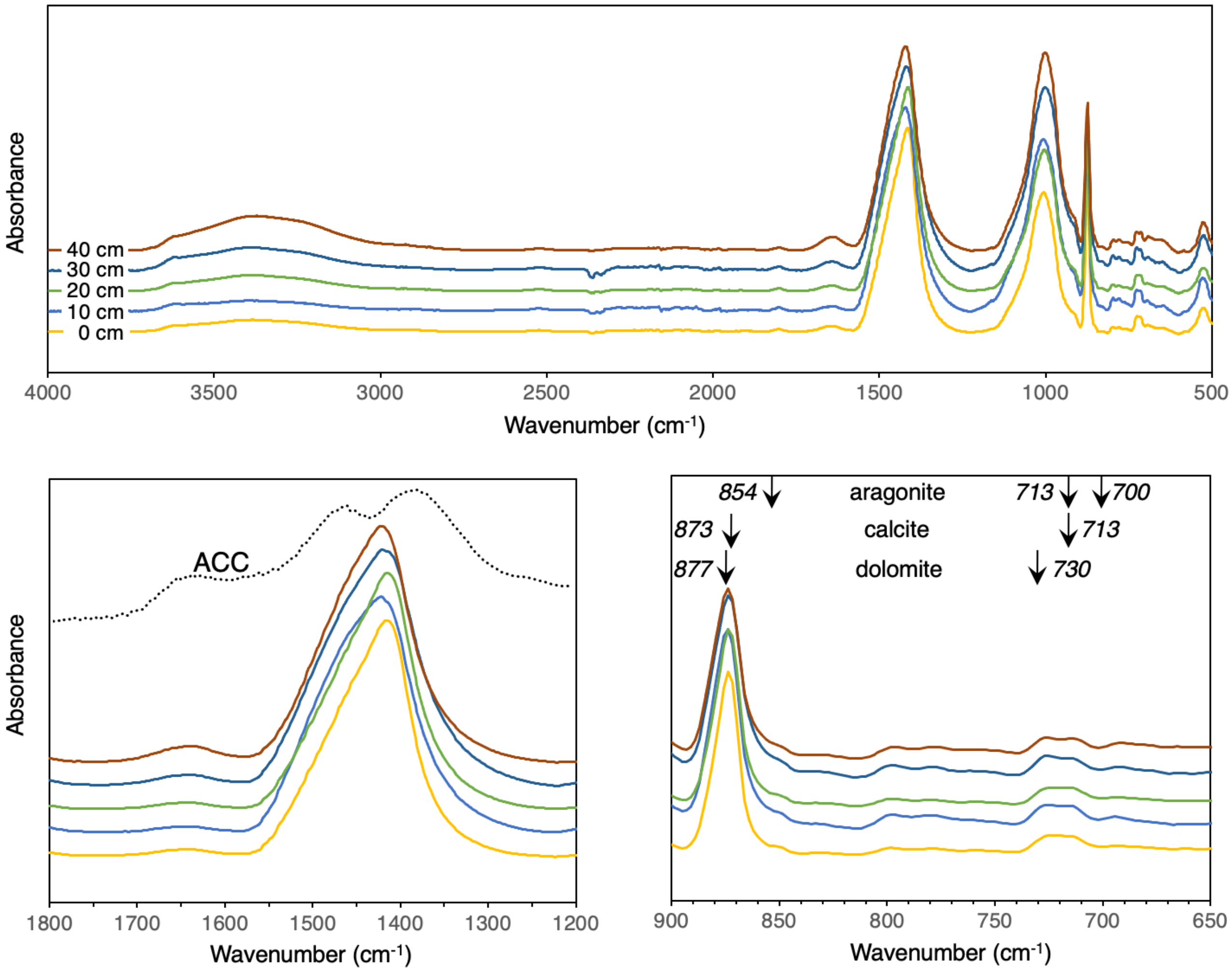

Fig. S3. Fourier-transform infrared spectroscopy spectra of the studied samples as well as a typical spectrum for amorphous calcium carbonate (ACC; after Demény et al., 2016). Characteristic peaks are indicated for aragonite, calcite and dolomite.

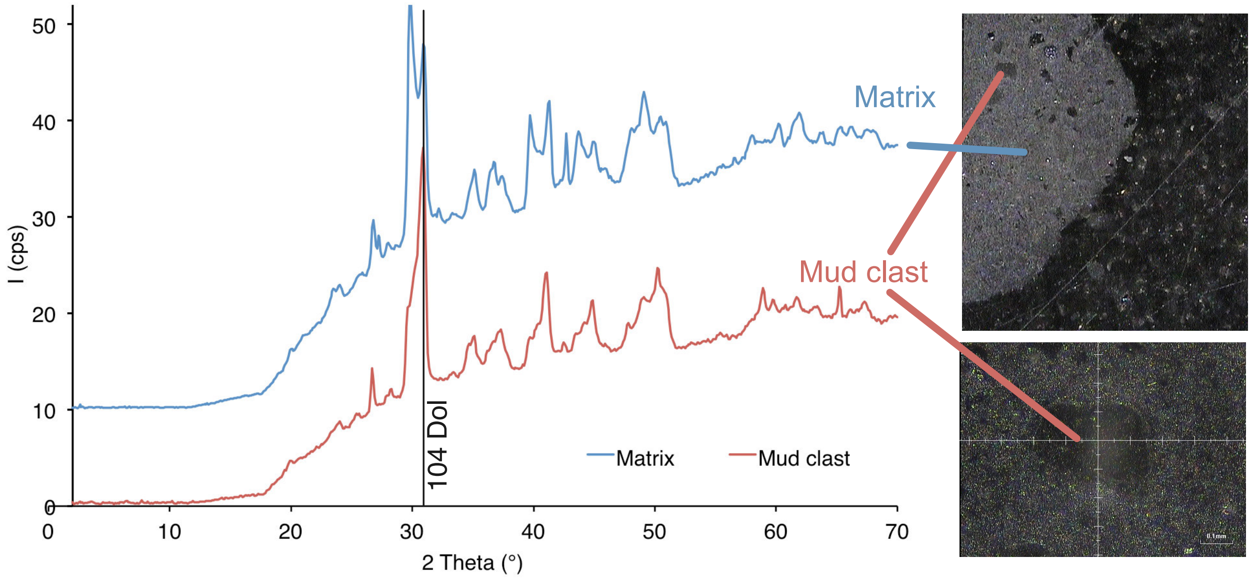

Fig. S4. Micro-XRD diagram of embedded bulk sediment reveals a 400-µm-sized mudclast of protodolomite embedded in a fine matrix of Mg-calcite and protodolomite.

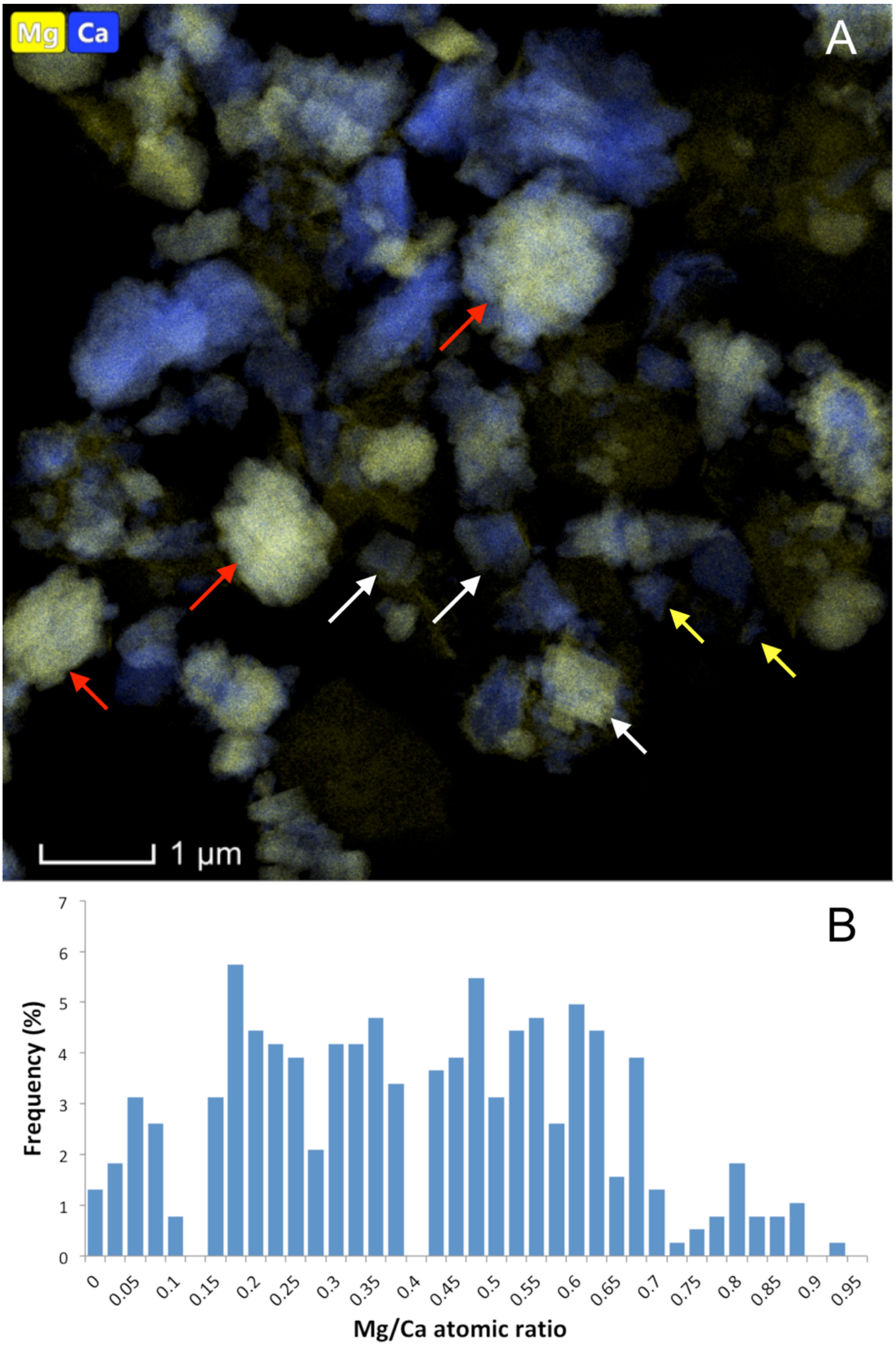

Fig. S5. (A) Elemental map of the <2 µm fraction analysed by EDX in the transmission electron microscope with scanning mode (STEM) revealed, besides low-Mg calcite, three different morphological types of Mg-Ca-carbonate crystals: rhombohedral crystals (white arrows), irregular crystals (yellow arrows), and polycrystalline-looking aggregates (red arrows). (B) Histogram showing abundance distribution of Mg/Ca atomic ratios of the measured carbonate grains, after correcting for the Mg content of the associated silicates. The same groups as detected by XRD occur, but with almost continuous transitions.

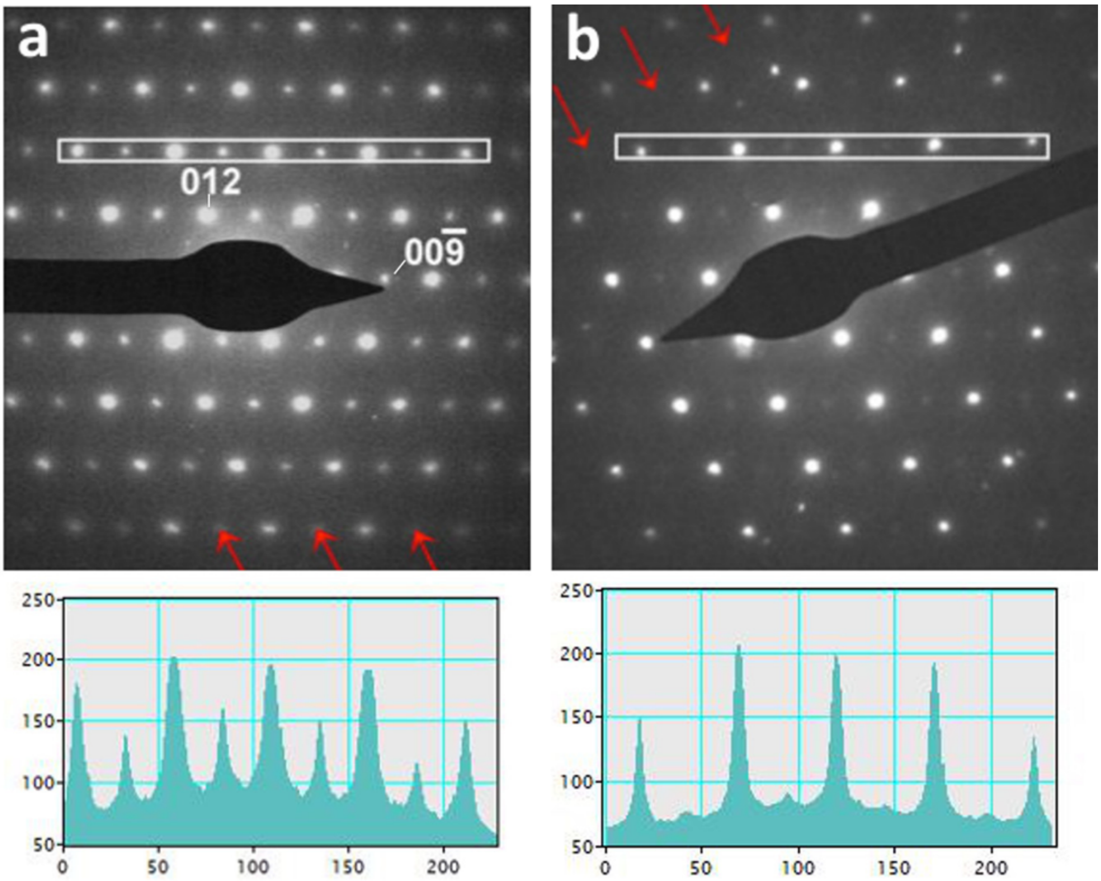

Fig. S6. Example of SAED patterns obtained from Mg-rich parts of carbonate grains, both showing ordering reflections along the rows marked by red arrows. The relative intensities of these reflections are much stronger in (a) than in (b), as shown by the intensity profiles (in the lower panels) obtained for the boxed row of spots, indicating a higher degree of ordering of Mg and Ca in (a) than in (b).
